# Global Epidemiology of Diphtheria, 2000–2017^1^

**DOI:** 10.3201/eid2510.190271

**Published:** 2019-10

**Authors:** Kristie E.N. Clarke, Adam MacNeil, Stephen Hadler, Colleen Scott, Tejpratap S.P. Tiwari, Thomas Cherian

**Affiliations:** Centers for Disease Control and Prevention, Atlanta, Georgia, USA (K.E.N. Clarke, A. MacNeil, S. Hadler, C. Scott, T.S.P. Tiwari);; World Health Organization, Geneva, Switzerland (T. Cherian)

**Keywords:** diphtheria, Corynebacterium diphtheria, bacteria, data quality, public health surveillance, global, epidemiology

## Abstract

In 2017, a total of 8,819 cases of diphtheria were reported worldwide, the most since 2004. However, recent diphtheria epidemiology has not been well described. We analyzed incidence data and data from the literature to describe diphtheria epidemiology. World Health Organization surveillance data were 81% complete; completeness varied by region, indicating underreporting. As national diphtheria–tetanus–pertussis (DTP) 3 coverage increased, the proportion of case-patients <15 years of age decreased, indicating increased protection of young children. In countries with higher case counts, 66% of case-patients were unvaccinated and 63% were <15 years of age. In countries with sporadic cases, 32% of case-patients were unvaccinated and 66% were >15 years of age, consistent with waning vaccine immunity. Global DTP3 coverage is suboptimal. Attaining high DTP3 coverage and implementing recommended booster doses are necessary to decrease diphtheria incidence. Collection and use of data on subnational and booster dose coverage, enhanced laboratory capacity, and case-based surveillance would improve data quality.

Diphtheria was a leading cause of childhood death in the prevaccine era (*1*). Incidence in industrialized countries decreased rapidly with diphtheria–tetanus–pertussis (DTP) vaccine introduction after World War II. Incidence in less developed countries also decreased after the launch of the World Health Organization (WHO) Expanded Programme on Immunization in 1974 (*2*), which recommended that all infants receive a 3-dose series of DTP vaccine by 6 months of age. A spike in incidence in the newly independent states of the former Soviet Union occurred in the 1990s (Figure 1), resulting in >157,000 cases and 5,000 deaths (*1*). This spike demonstrated the potential for severe outbreaks of diphtheria in communities that have a large population of nonimmune adults and poor vaccination coverage for children.

**Figure 1:**
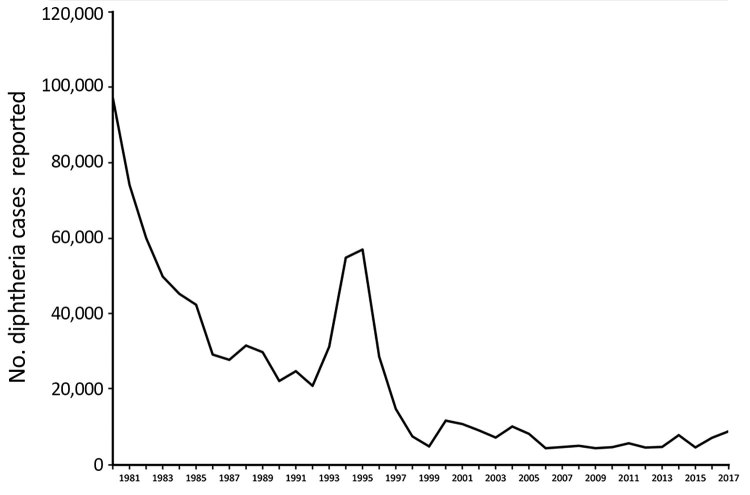
Cases of diphtheria as reported to the World Health Organization and the United Nations Children’s Fund, through the Joint Reporting Form, worldwide, 1980−2017.

Although several comprehensive reviews were published after that outbreak peaked (*3**–**5*), only sporadic documentation of diphtheria outbreaks has been published since, and no examination of global epidemiologic trends has been published. During 2016–2019, diphtheria outbreaks were reported in multiple countries, including Bangladesh, Yemen, and Venezuela. Several outbreaks were among vulnerable populations or in areas of social disruption and conflict. Authors in some low- and middle-income countries have reported a resurgence of the disease or a shift to older populations (*6**–**8*). However, the quality of reported surveillance data varies; 26 of 130 responding countries report no diphtheria surveillance system, and only 55 report case-based surveillance with laboratory confirmation (*9*). In this context, a review of recent epidemiologic trends is needed to better characterize recent outbreaks.

Given a previous lack of global guidance on diphtheria-containing booster doses after the 3-dose primary series, a wide variety of schedules had been adopted by different countries as of 2018 (*10**–**12*). Twenty-four percent of countries used the 3-dose series alone, and other countries offered 1–3 booster doses on varying schedules; 24% of countries also included >1 adult booster doses, defined as a dose recommended at or after 18 of years of age (Figure 2). Although there are no global estimates of coverage for booster doses, available data suggest coverage is lower than that for the primary series in many countries (*13*).

**Figure 2:**
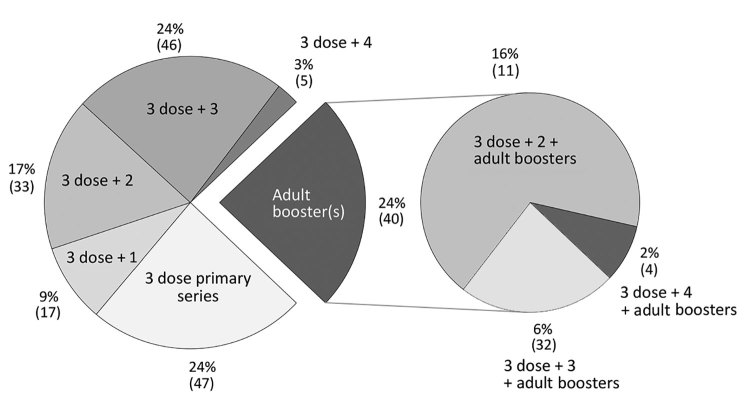
Percentage (number) of countries reporting each diphtheria vaccination schedule, 2018. The number after the plus sign indicates the number of booster doses on the national schedule after the 3-dose primary series and before the age of 18 years.

In August 2017, WHO released revised recommendations for diphtheria vaccination (*14*). In addition to the 3-dose primary series in infancy, new recommendations include 3 diphtheria toxoid–containing booster doses given at 12–23 months of age, 4–7 years of age, and 9–15 years of age. These recommendations, which harmonize with the updated recommendations for tetanus boosters released in February 2017 (*15*), emphasize the need for a life course vaccination approach and present new opportunities for synergies with other vaccines and healthcare activities, such as measles second dose, preventive care at school entry, and human papillomavirus vaccination. In addition, it is now recommended that the combined tetanus toxoid and diphtheria toxoid vaccine be used during pregnancy and when tetanus prophylaxis is required because of injury, rather than using tetanus toxoid alone. The objective of this study was to review the epidemiology of diphtheria since 2000, including global aggregate surveillance data, vaccination coverage data, and available data regarding the age and vaccination status of infected persons.

## Methods

We examined aggregate diphtheria surveillance data reported annually to WHO and the United Nations Children’s Fund (UNICEF) from each country through the Joint Reporting Form (JRF) for global and regional epidemiologic incidence trends during 2000–2017. JRF data include the aggregate number of cases of diphtheria reported by countries in a given year and do not provide information on case-patient age or vaccination status.

We obtained information on the age or vaccination status of diphtheria case-patients from accessible published or gray literature (publications produced by organizations outside traditional commercial or academic publishing and distribution channels), presentation reports, and regional case-based surveillance data. We performed systematic searches covering publication dates during January 2000–September 2018 (Table 1). Searches returned 1,080 unique abstracts; 2 researchers reviewed each abstract to determine relevance. Discrepancies were discussed until consensus was reached. To meet inclusion criteria, manuscripts had to contain data about the age or vaccination status of case-patients with respiratory diphtheria caused by *Corynebacterium diphtheriae* during 2000–2017, with availability of full text in English or Spanish. Twenty-nine abstracts were excluded for language, as were 779 abstracts not relevant to the review. Two additional articles were not retrievable in full text.

**Table 1:** Strategy for systematic literature search for diphtheria, January 1, 2000-September 18, 2018*

Database	Initial search strategy
Medline	(Diphtheria/ AND Disease Outbreaks/) OR (diphtheria.ti AND (outbreak* OR cluster* OR epidemic*).ti,ab.) OR (diphtheria ADJ3 (outbreak* OR cluster* OR epidemic*)).ab.
Embase	(Diphtheria/ AND Disease Outbreaks/) OR (diphtheria.ti AND (outbreak* OR cluster* OR epidemic*).ti,ab.) OR (diphtheria ADJ3 (outbreak* OR cluster* OR epidemic*)).ab.
Scopus	TITLE-ABS-KEY(diphtheria W/2 outbreak*)
Database	Secondary Search Strategy
Medline	*diphtheria/ or diphtheria.ti,ab. AND Epidemics/ OR Disease Outbreaks/ OR (outbreak* OR cluster* OR epidemic*).ti,ab. AND Limit 2000–
Embase	*diphtheria/ or diphtheria.ti,ab. AND Epidemic/ OR (outbreak* OR cluster* OR epidemic*).ti,ab. AND Limit 2000–
Global Health	diphtheria/ OR diphtheria.ti,ab,sh. AND Epidemics/ OR (outbreak* OR cluster* OR epidemic*).ti,ab,sh. AND Limit 2000–
CINAHL	(MJ diphtheria) or (TI diphtheria) OR (AB diphtheria) AND (MH “Disease Outbreaks”) OR (MH Epidemics) OR (TI (outbreak* OR cluster* OR epidemic*)) OR (AB (outbreak* OR cluster* OR epidemic*)) AND Limit 2000- ; Exclude Medline records
Cochrane Library	[mh diphtheria] or diphtheria:ti,ab AND [mh “Disease Outbreaks”] OR [mh Epidemics] OR (outbreak* OR cluster* OR epidemic*):ti,ab AND Limit 2000–
LILACS	Diphtheria AND (outbreak* OR cluster* OR epidemic*)
Scopus	INDEXTERMS(Diphtheria) AND INDEXTERMS(“disease outbreak*” OR epidemic*) AND (LIMIT-TO(PUBYEAR,2015) OR LIMIT-TO(PUBYEAR,2014) OR LIMIT-TO(PUBYEAR,2013) OR LIMIT-TO(PUBYEAR,2012) OR LIMIT-TO(PUBYEAR,2011) OR LIMIT-TO(PUBYEAR,2010) OR LIMIT-TO(PUBYEAR,2009) OR LIMIT-TO(PUBYEAR,2008) OR LIMIT-TO(PUBYEAR,2007) OR LIMIT-TO(PUBYEAR,2006) OR LIMIT-TO(PUBYEAR,2005) OR LIMIT-TO(PUBYEAR,2004) OR LIMIT-TO(PUBYEAR,2003) OR LIMIT-TO(PUBYEAR,2002) OR LIMIT-TO(PUBYEAR,2001) OR LIMIT-TO(PUBYEAR,2000)) AND (LIMIT-TO(DOCTYPE,”ar”) OR LIMIT-TO(DOCTYPE,”re”)) AND (LIMIT-TO(EXACTKEYWORD,”Diphtheria”))

*CINAHL**,** Cumulative Index to Nursing and Allied Health Literature; LILACS**,** Latin American and Caribbean Health Sciences Literature**.**

Of 107 manuscripts reviewed in full text, 28 met inclusion criteria for the analysis (Appendix references *1–28*). The full text of each manuscript was reviewed by 2 investigators, and relevant data were compiled in an Excel (Microsoft, https://www.microsoft.com) database. An additional 19 published manuscripts were identified through the reference lists (Appendix references *29–47*). A review of the gray literature resulted in 12 additional sources (Appendix references *48–59*), and communications with colleagues resulted in access to 11 unpublished reports (Pan American Health Organization: T.S.P. Tiwari [2]; R. Kaiser; Centers for Disease Control and Prevention; WHO Punjab; J. Crucena; Republic of Philippines Epidemiology Bureau; T. Nguyen; A. Nihal; and L. Sangal).

Diphtheria data from the European Surveillance System were provided by Spain, Latvia, Germany, Italy, Lithuania, the Netherlands, the United Kingdom, Finland, Sweden, France, Austria, and Belgium and released by the European Centre for Disease Prevention and Control (Stockholm, Sweden) (Appendix reference *60*). Similar case-based diphtheria data were not available from other regions. Because of multiple data sources, we conservatively excluded cases identified as potential duplicates when matching by age group, location, and year. The final dataset consisted of 15,380 cases of diphtheria (15,068 including age data and 7,242 including vaccination status data) from 34 countries.

We compared cases included in the final dataset with the number of cases in the aggregate JRF data for each country over the same period to cross-reference the completeness of the diphtheria data reported in the JRF. Because DTP 3 coverage is a major risk factor for disease transmission in a population, we took the average of the national WHO–UNICEF estimates of DTP3 coverage (*16*) for the 5 years previous to the cases for each set of reported cases. For analysis, we classified countries with data included in the review as either countries with higher case counts (defined as reporting >10 cases in each of >3 years of JRF incidence data during 2000–2017, or reporting >100 cases in a single year) or as countries with sporadic cases.

The age distribution analysis was complicated by the diverse ways in which age data were aggregated in different manuscripts. Our analysis used an age of 15 years for disaggregation of age data because this age was most frequently mentioned in the historical literature as a benchmark for the age shift in diphtheria incidence over time. However, on the basis of availability of age data from source documents, we made classifications by using heterogeneous age cutoffs in the initial analysis. To address this limitation, we compiled a more precise dataset (n = 9,334 cases from 32 countries) for sensitivity analyses that contained only data that classified case-patients as >15 or <15 years of age (± 1 year).

Sources also aggregated vaccination status data differently and had varying definitions for fully vaccinated depending on the vaccination schedule of the country or criteria of the investigators. For this review we defined fully vaccinated, at a minimum, as having received all 3 doses of the primary series. Cases with partial vaccination (>1 dose of infant DTP) were grouped with fully vaccinated cases in several sources; we conservatively designated these cases as partially vaccinated in the full dataset. Reports of cases with unknown or partial vaccination status were grouped with unvaccinated cases in other sources; we conservatively designated these cases as unvaccinated in the full dataset. To address this limitation, we compiled a dataset (1,534 cases from 27 countries) that contained only cases that were reported in >3 different groups (unvaccinated, partially vaccinated, or fully vaccinated). We also provide additional information on datasets compiled from the literature and gray literature review (Table 2).

**Table 2:** Overview of datasets with information on age and vaccination status compiled from review compared with aggregate diphtheria incidence data reported by countries on the joint reporting form over the same period, 2000–2017*

Country	Classification	Full dataset, n = 15,380			Datasets for sensitivity analysis							
					Age data, n = 9,334†			Vaccination status data, n = 1,534‡			Joint reporting form data 2000–2017, n = 103,138	
		Years of data	Total cases		Years of data	Total cases		Years of data	Total cases		Years ≥1 case reported	Total cases
Afghanistan	Higher case count	1	50		1	37		NA	NA		8	1,380
Australia	Sporadic	1	1		1	1		1	1		7	26
Austria	Sporadic	1	2		1	2		NA	NA		2	4
Bangladesh§	Higher case count	1	3,581		1	3,567		NA	NA		18	804
Belgium	Sporadic	2	2		2	2		1	1		7	15
Brazil	Higher case count	3	32		2	28		3	32		16	331
Colombia	Sporadic	1	8		1	8		1	6		4	17
Dominican Republic	Higher case count	2	82		2	82		1	1		15	372
Finland	Sporadic	2	2		2	2		2	2		3	3
France	Sporadic	7	22		7	22		4	4		10	54
Germany	Sporadic	7	24		7	24		3	3		13	77
Haiti	Higher case count	6	314		3	92		3	41		14	230
India¶	Higher case count	20	8,720		18	3,303		12	544		18	79,034
Indonesia	Higher case count	2	582		2	566		1	52		17	7,160
Italy	Sporadic	1	1		1	1		NA	NA		1	1
Laos	Higher case count	2	62		2	62		2	27		15	578
Latvia	Higher case count	10	133		10	133		6	45		18	612
Lithuania	Sporadic	2	3		2	3		NA	NA		4	10
Malaysia	Higher case count	1	1		1	1		NA	NA		14	106
Myanmar	Higher case count	2	156		2	154		1	50		17	512
Netherlands	Sporadic	3	5		3	5		3	4		5	12
Nigeria	Higher case count	6	118		6	118		3	8		4	7,565
Norway	Sporadic	3	8		3	8		1	3		3	5
Pakistan	Higher case count	2	406		2	406		2	372		18	1,176
Paraguay	Sporadic	1	47		NA	NA		1	12		5	59
Philippines	Higher case count	7	553		7	512		6	280		15	1,019
South Africa	Sporadic	1	15		1	15		1	8		6	26
Spain	Sporadic	2	2		2	2		2	2		2	2
Sweden	Sporadic	6	12		6	12		5	7		6	12
Thailand	Higher case count	2	47		2	35		NA	NA		18	342
United Kingdom	Sporadic	8	23		8	22		4	4		13	40
United States	Sporadic	1	1		1	1		1	1		5	6
Venezuela	Higher case count	2	244		NA	NA		1	11		3	818
Vietnam	Higher case count	2	121		1	108		1	13		18	730

*NA, not available. †Includes all case-patients for which age was clearly designated as above or below an age cutoff point of 15 y (± 1 y). ‡Includes all cases for which vaccination status was clearly designated as fully vaccinated, partially vaccinated, or unvaccinated. §Population of Rohingya refugees from Myanmar housed in a refugee camp in Bangladesh. ¶Data from India include 2 articles with aggregate data including the late 1990s (Appendix references *15,18*).

We analyzed distribution of case-patients by age and vaccination status by using descriptive methods in Excel 2016. We performed sensitivity analysis to test for consistency of findings between those analyses performed by using the full dataset and those performed by using a dataset with enhanced precision around the variable of interest. Because cases from India represented >50% of cases in the full dataset, we conducted a final sensitivity analysis to check for consistency of trends for all analyses when data from India were excluded.

Finally, to examine the relationship between vaccination coverage with the primary series and age distribution, we combined the total number of cases for each country in the dataset. We compared the proportion of case-patients >15 years of age in each country with the average of DTP3 coverage for the 5 years preceding the year(s) when the cases were reported. We excluded countries with <5 cases in the dataset from this analysis, leaving 24 countries in the primary analysis and 23 countries in the sensitivity analysis.

## Results

### General Epidemiologic Trends

**Figure 3:**
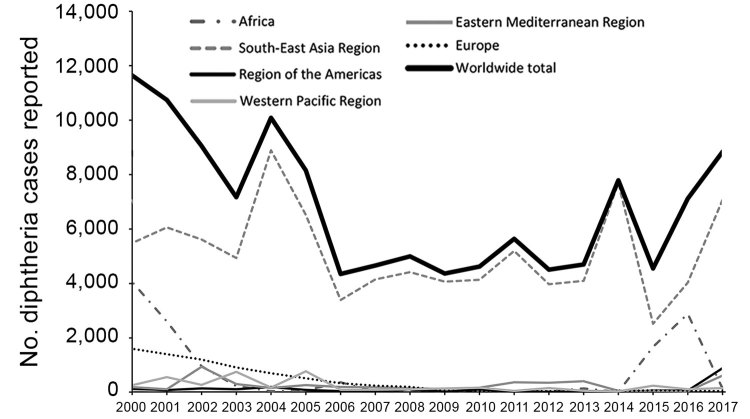
Reported cases of diphtheria per Joint Reporting Form, by World Health Organization region and worldwide, 2000−2017.

Since 2000, the number of reported diphtheria cases worldwide in JRF data initially decreased, then leveled at 4,300–5,700 reported cases/year during 2006–2013. Subsequently, the annual number of reported cases became more variable; 8,819 cases were reported in 2017, the most cases in a single year since 2004 (Figure 3). The average number of annual cases reported worldwide over the most recently reported 5-year period (2013–2017) was 6,582, an increase of 37% compared with the previous 5-year average of 4,809 cases during 2008–2012.

Since 2000, the WHO South-East Asia region has reported most of the global diphtheria incidence each year. India has reported the largest proportion of diphtheria cases in aggregate JRF data since 2000 (64%); similarly, in data compiled from the literature review, >50% of cases with age and vaccination status were from India in the full dataset (8,720 [57%]). Collectively, India, Nepal, and Indonesia have reported 96%–99% of the cases in the South-East Asia region since 2000. Meanwhile, cases reported from the WHO Europe region decreased as the impact of the large outbreak in the former Soviet Republics during the 1990s attenuated.

### Surveillance Data Completeness and Accuracy

During 2000–2017, each country (except South Sudan) had the opportunity to submit 18 years of JRF data on diphtheria incidence to WHO, which provided a maximum of 3,481 potential country-years of data. Although these surveillance data are known to have limitations (*17*), they represent the most complete existing database for worldwide disease incidence. However, 19% of country-years were missing globally. Missing JRF diphtheria data were not equally distributed among regions. The Africa region had the highest percentage of missing country-years (40%); this percentage included substantial periods of missing data from populous countries, including Nigeria (66% of country-years missing data), Kenya (78%), Uganda (89%), and Ethiopia (100%). The Western Pacific (22%) and Eastern Mediterranean (22%) regions also had an above average proportion of missing country-years. In the remaining regions, 2%–11% of country-years were missing.

We cross-referenced the years and countries with cases in the full review dataset with JRF data. Overall, when data were cross-checked between aggregate JRF data and case data compiled from manuscripts and outbreak reports, we identified 36 instances in which diphtheria data reported through the JRF were inconsistent with those reported in the published literature. In 20 instances, we found case data during the review from countries with missing JRF data for diphtheria in the corresponding year(s); in 7 instances, countries had reported 0 cases for the corresponding year(s); and in 9 additional instances, the number of cases found in the review exceeded the number reported in the JRF.

### Vaccination Status of Diphtheria Case-Patients

Analysis showed that 65% of case-patients in the full dataset were unvaccinated, 13% were partially vaccinated, and 22% were vaccinated with >3 doses of diphtheria toxoid–containing vaccine. In a sensitivity analysis that only included cases that had more precise data on vaccination status, we found that the proportion of unvaccinated case-patients increased to 72%.

**Figure 4:**
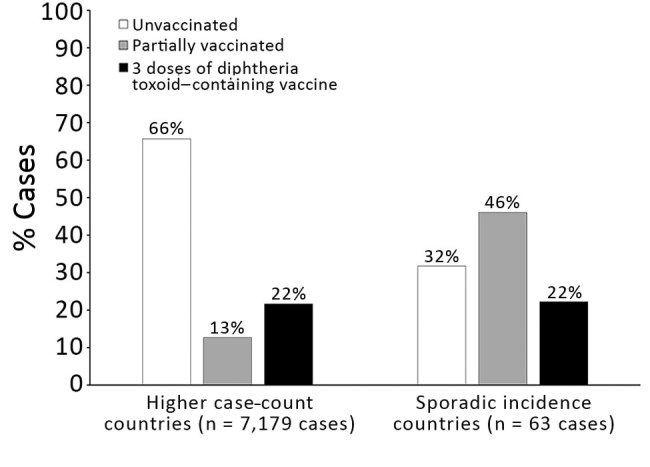
Vaccination status of diphtheria cases in higher case count versus sporadic incidence countries (full dataset, 34 countries), 2000–2017.

In countries with higher case counts, most case-patients were unvaccinated (Figure 4). Among case-patients with known vaccination status in the full review dataset (n = 7,242), 66% were unvaccinated in higher case count countries; this percentage was 73% in the sensitivity analysis restricted to case-patients with precise vaccination status data (n = 1,534). Excluding cases from India showed that these percentages were similar (63% in the primary dataset and 66% in the sensitivity analysis dataset). In countries with sporadic incidence, vaccination status of case-patients was more evenly distributed; the largest proportion was in the partially vaccinated category in both the main analysis (46%) and the sensitivity analysis (38%).

### Age of Diphtheria Case-Patients

In the dataset overall (15,068 case-patients with age data), 37% of case-patients were >15 years of age. In a sensitivity analysis of the dataset with more precise age data, we found that 34% were >15 years of age.

**Figure 5:**
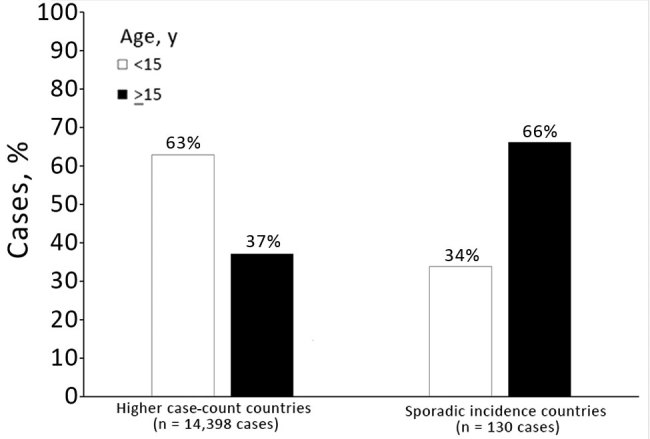
Proportion of diphtheria case-patients <15 years and >15 years of age in higher case count versus sporadic incidence countries (full dataset, 34 countries), 2000–2017.

Proportions of case-patients >15 years of age differed markedly between countries with sporadic incidence and those with higher case counts (Figure 5); this finding was consistent across the primary and sensitivity analyses. In higher case-count countries, there was a lower proportion of cases >15 years of age when examined in the full dataset (37%) and the dataset with more precise age data (34%). When data from India were further excluded among higher case count countries, there was an even lower proportion of case-patients >15 years of age in the main dataset (25%) and the dataset with more precise age data (34%). Conversely, in sporadic incidence countries, 66% of the case-patients were >15 years of age in the full dataset and on sensitivity analysis.

### Relationship between Age of Case-Patients and Vaccination Coverage

**Figure 6:**
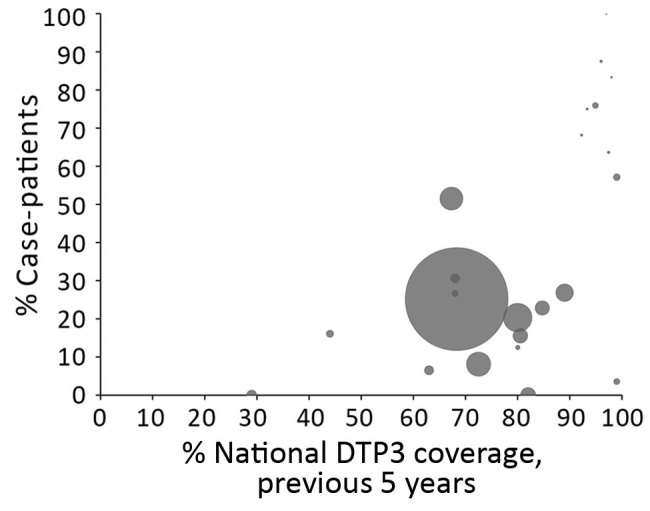
Percentage of diphtheria case-patients >15 years of age, by national DTP3 coverage, 2000–2017. Each circle represents a country, and its size is proportionate to the average number of cases reported from the country per year of data in the dataset. The largest data point represents a large number of cases in a single year among Rohingya refugees from Myanmar. The vaccination coverage of the Rohingya population is unknown; therefore, the average of DTP3 coverage of Rakhine State in Myanmar from 2016–2017 was used (*18*). DTP3, diphtheria–tetanus–pertussis vaccine; UNICEF, United Nations Children’s Fund; WHO, World Health Organization.

When we examine the relationship between vaccination coverage with the primary series and age distribution, we found a visible trend toward a higher percentage of case-patients >15 years of age in countries with higher DTP3 coverage in both the full dataset (Figure 6) and a sensitivity analysis on the dataset with more precise age data. In particular, in almost all countries with DTP3 coverage >90%, >50% of diphtheria cases occurred among persons >15 years of age.

## Discussion

It is clear from aggregate incidence data that progress in decreasing diphtheria incidence worldwide has stalled, and reported cases have recently increased. Larger societal factors, such as population migration or political instability, can create conditions favorable to an outbreak; the largest recent outbreaks (January 2016–February 2019) were reported in the Rohingya refugee population in Bangladesh (8,403 cases), as well as areas experiencing conflict or social disruption, such as Yemen (3,340 cases) and Venezuela (2,512 cases) (*19*). The South-East Asia region has reported most of the diphtheria cases since 2000, which might be caused by the large populations of several countries in the region that have endemic disease. However, incomplete data from other regions could be obscuring additional major foci of disease incidence. Global and regional trends can be shaped by incomplete data, including years of nonreporting or underreporting by populous and high-incidence countries.

The diphtheria data reported annually to WHO and UNICEF on the JRF have substantial limitations in terms of quality and reflect opportunities to improve disease surveillance. We found these data to be incomplete when cross-referenced with the literature, indicating a likely underestimate of incidence worldwide and a decreased understanding of the burden of disease. However, in some countries with lower laboratory capacity, only a small proportion of cases are laboratory confirmed, which could result in overreporting in some settings (*19*). The ability to use aggregate data for action is limited by the lack of key variables, including vaccination status, age, and subnational location.

Implementation of case-based surveillance for diphtheria, combined with availability of subnational coverage data, would result in improved understanding of diphtheria epidemiology and enhanced capability to prevent and respond to outbreaks. A 2017 gap analysis of diphtheria diagnostic capacity in the European Union found substantial gaps, including lack of sufficient laboratory systems with methods to determine toxigenicity, difficulty obtaining primary media culture, and challenges to obtaining diphtheria antitoxin for both laboratory diagnosis and clinical management of cases (*20*). Similar assessments are currently being conducted to more fully understand the scope of challenges in other regions (A. Efstratiou, National Infection Service, London, UK, pers. comm., 2019 Aug 1). Worldwide, only 55 countries report conducting national, case-based surveillance for diphtheria with laboratory confirmation (*9*). In response to the limitations of current disease surveillance systems, WHO has released new comprehensive surveillance recommendations (*21*). For diphtheria, immediate investigation and collection of case-based data are recommended for all outbreaks. These guidelines provide a set of recommended minimum data elements to collect, as well as recommended analyses and uses of data collected. These standards represent an opportunity to improve and standardize available data with widespread implementation but will require investments in both surveillance and laboratory capacity.

Accounts of outbreaks in the peer-reviewed literature, gray literature, presentations, and other reports were compiled as the best available sources of information on case-patient age and vaccination status. When examining these data, we found that most diphtheria cases occur in unvaccinated persons, particularly in countries with higher case counts where most disease is among children <15 years of age. Therefore, achieving adequate coverage with the primary series is urgently needed. Ensuring high primary series coverage is especially useful in the context of vaccine hesitancy, which has wide variability among countries and regions (*22*). DTP3 coverage worldwide has stagnated at 84%–85% since 2010 (*23*), and improving this coverage through increased equity of and access to routine immunization services is key to the efforts to combat diphtheria. Countries with sporadic incidence of diphtheria have a more even distribution of vaccination status among cases. Age data from these countries also reflect a higher proportion of cases in the adolescent and adult populations. The predominance of older case-patients in these countries, taken together with the higher proportion of case-patients who have received, at a minimum, 3 doses of diphtheria-containing vaccine in infancy, indicate that waning immunity is also a major issue. This issue can be addressed through widespread adoption of the 3 diphtheria toxoid–containing booster doses recommended in 2017 by WHO and recently approved as a future investment in the 2021–2025 Gavi Vaccine Investment Strategy (*24*).

In our dataset, as vaccination coverage in a country increased, the percentage of case-patients >15 years of age also increased. This increase in the proportion of older case-patients is not unique to diphtheria because similar changes have been seen in the epidemiology of other vaccine-preventable diseases as coverage increased (*25*). This finding indicates that the large proportion of case-patients >15 years of age in countries with sporadic incidence probably represents a proportional, rather than an absolute, increase because high vaccination coverage in childhood resulted in fewer susceptible persons in this age group. Many adults might have grown up during a time when no or fewer booster doses were given, although information on historical changes to vaccination schedules is incomplete. As countries implement or modify booster dose schedules according to the new WHO recommendation, data on booster dose coverage would improve understanding of susceptibility to disease in different age groups.

Limitations of this analysis include heterogeneous methods used by available sources to aggregate data on case-patient age and vaccination status. Because the available data might not be representative, findings might not be generalizable to all contexts. Because of lack of laboratory capacity in some settings, many cases in the literature are not confirmed by culture. Strengths of the analysis include the compilation of all known available data on the age and vaccination status of diphtheria case-patients, which highlight that data from published disease outbreaks are a useful resource for describing epidemiologic changes and to triangulate with other existing data sources. Limitations of the dataset were addressed as comprehensively as possible through sensitivity analyses of subsets of cases with more precise data to validate trends observed on analysis of the full dataset.

In light of a recent increase in reported cases, action is needed to make progress in combating diphtheria. However, many national immunization schedules lag behind current recommendations, and the lack of case-based diphtheria data limits the ability to take targeted action. Intensified efforts to improve routine immunization coverage with DTP3 and to implement recommended booster doses would help to decrease diphtheria cases by both decreasing the susceptibility of children and addressing the problem of waning immunity among adolescents and adults. Implementing new WHO guidelines (*21*) would result in case-based diphtheria surveillance data with laboratory confirmation that could be analyzed by using standardized age categories. This implementation would greatly enhance available data and highlights the need for enhanced laboratory capacity to provide these case confirmations, particularly in countries with endemic disease and other lower-middle-income and low-income countries. Increased availability of booster dose coverage data, subnational coverage data, and more complete historical records of changes to immunization schedules would lend context to data on incidence and epidemiologic trends. An improvement in the quality and consistency of data collected on diphtheria would create a stronger evidence base for future research, timely interventions, and recommendations to combat this deadly disease.

Appendix Literature review references on global epidemiology of diphtheria, 2000–2017.
