# Supplementary material for: Global Epidemiology of Diphtheria, 2000–2017
Source: Emerg Infect Dis. 2019 Oct;25(10):1834–42. doi: 10.3201/eid2510.190271 (PMC6759252; doi:10.3201/eid2510.190271)
Supplement: Appendix — Literature review references on global epidemiology of diphtheria, 2000–2017. [file 19-0271-Techapp-s1.pdf]

Article DOI: https://doi.org/10.3201/eid2510.190271

Global Epidemiology of Diphtheria, 2000–2017

Appendix

References gathered by review of published and gray literature (publications produced by organizations outside traditional commercial or academic publishing and distribution channels)

References

1. Dravid MN, Joshi SA. Resurgence of diphtheria in Malegaon and Dhule regions of north Maharashtra. Indian J Med Res. 2008;127:616–7. PubMed

2. Landazabal García N, Burgos Rodríguez MM, Pastor D. Diphtheria outbreak in Cali, Colombia, August–October 2000. Epidemiol Bull. 2001;22:13–5. PubMed

3. Quarterly communicable disease review January to March 2002. J Public Health Med. 2002;24:232–8. PubMed https://doi.org/10.1093/pubmed/24.3.232

4. Disease watch. Expert Rev Anti Infect Ther. 2003;1:355. https://doi.org/10.1586/14787210.1.3.355

5. Anima H, Malay M, Santanu H, Rajashree R, Sita C, Baran SA. A study on determinants of occurrence of complications and fatality among diphtheria cases admitted to ID and BG Hospital of Kolkata. J Commun Dis. 2008;40:53–8. PubMed

6. Bonmarin I, Guiso N, Le Flèche-Matéos A, Patey O, Patrick AD, Levy-Bruhl D. Diphtheria: a zoonotic disease in France? Vaccine. 2009;27:4196–200. PubMed https://doi.org/10.1016/j.vaccine.2009.04.048

7. Nath B, Mahanta TG. Investigation of an outbreak of diphtheria in Borborooah Block of Dibrugarh District, Assam. Indian J Community Med. 2010;35:436–8. PubMed https://doi.org/10.4103/0970-0218.69282

8. Perkins S, Cordery R, Nixon G, Abrahams A, Andrews J, White J, et al. Investigations and control measures following a non-travel-associated case of toxigenic *Cornyebacterium diphtheriae*, London, United Kingdom, December 2009-January 2010. Euro Surveill. 2010;15:pii:19544. PubMed

9. Saikia L, Nath R, Saikia NJ, Choudhury G, Sarkar M. A diphtheria outbreak in Assam, India. Southeast Asian J Trop Med Public Health. 2010;41:647–52. PubMed

10. Chumachenko T, Podavalenko A, Glushkevich T. Spread of *Corynebacterium diphtheriae* in Ukraine during post-epidemic period. Clinical Microbiology and Infection. 2011;17:S285.

11. Jalani NT, Mapue M, Lopez J, Tayag E, editors. Diphtheria outbreak in an urban city in central Philippines, 2010. EIS Conference International Night; 2011; Atlanta: Centers for Disease Control and Prevention; 2011 [cited 2019 Jul 26]. https://www.tephinet.org/diphtheria-outbreak-in-an-urban-city-in-central-philippines-2010

12. Niti T, Jignesh C, Himanshu N, Chintul S, Krunal M, Jaydeep D. Investigation of an outbreak of diphtheria in Dabela Village of Amirgagh Taluka and CHC, Banaskantha, Gujarat. National Journal of Community Medicine. 2011;2:196–200.

13. Rasmussen I, Wallace S, Mengshoel AT, Høiby EA, Brandtzaeg P. Diphtheria outbreak in Norway: lessons learned. Scand J Infect Dis. 2011;43:986–9. PubMed https://doi.org/10.3109/00365548.2011.600326

14. Besa NC, Coldiron ME, Bakri A, Raji A, Nsuami MJ, Rousseau C, et al. Diphtheria outbreak with high mortality in northeastern Nigeria. Epidemiol Infect. 2014;142:797–802. PubMed https://doi.org/10.1017/S0950268813001696

15. Phalkey RK, Bhosale RV, Joshi AP, Wakchoure SS, Tambe MP, Awate P, et al. Preventing the preventable through effective surveillance: the case of diphtheria in a rural district of Maharashtra, India. BMC Public Health. 2013;13:317. PubMed https://doi.org/10.1186/1471-2458-13-317

16. Wanlapakorn N, Yoocharoen P, Tharmaphornpilas P, Theamboonlers A, Poovorawan Y. Diphtheria outbreak in Thailand, 2012; seroprevalence of diphtheria antibodies among Thai adults and its implications for immunization programs. Southeast Asian J Trop Med Public Health. 2014;45:1132–41. PubMed

17. Rashid ZZ, Mohamed NA, Fong TS. A case of fatal diphtheria in a paediatric patient. Internet J Microbiol. 2015;14:1–5 [cited 2019 Jul 16]. http://ispub.com/IJMB/14/1/32981

18. Santos LS, Sant’anna LO, Ramos JN, Ladeira EM, Stavracakis-Peixoto R, Borges LLG, et al. Diphtheria outbreak in Maranhão, Brazil: microbiological, clinical and epidemiological aspects. Epidemiol Infect. 2015;143:791–8. PubMed https://doi.org/10.1017/S0950268814001241

19. Doanh PV, Pham TD, Vu NL, Chu VT, Do TH, Nguyen TT, et al. An outbreak of diphtheria in K'Bang District, Gia Lai, Vietnam, October 2013–July 2014. Int J Infect Dis. 2016;45:172. https://doi.org/10.1016/j.ijid.2016.02.406

20. M M, M R. Diphtheria in Andhra Pradesh: a clinical-epidemiological study. Int J Infect Dis. 2014;19:74–8. PubMed https://doi.org/10.1016/j.ijid.2013.10.017

21. Jané M, Vidal MJ, Camps N, Campins M, Martínez A, Balcells J, et al. A case of respiratory toxigenic diphtheria: contact tracing results and considerations following a 30-year disease-free interval, Catalonia, Spain, 2015. Euro Surveill. 2018;23. PubMed https://doi.org/10.2807/1560-7917.ES.2018.23.13.17-00183

22. Mahomed S, Archary M, Mutevedzi P, Mahabeer Y, Govender P, Ntshoe G, et al. An isolated outbreak of diphtheria in South Africa, 2015. Epidemiol Infect. 2017;145:2100–8. PubMed https://doi.org/10.1017/S0950268817000851

23. Das PP, Patgiri SJ, Saikia L, Paul D. Recent outbreaks of diphtheria in Dibrugarh District, Assam, India. J Clin Diagn Res. 2016;10:DR01–03. PubMed https://doi.org/10.7860/JCDR/2016/20212.8144

24. Parande MV, Roy S, Mantur BG, Parande AM, Shinde RS. Resurgence of diphtheria in rural areas of North Karnataka, India. Indian J Med Microbiol. 2017;35:247–51. PubMed

25. Jain A, Samdani S, Meena V, Sharma MP. Diphtheria: It is still prevalent!!! Int J Pediatr Otorhinolaryngol. 2016;86:68–71. PubMed https://doi.org/10.1016/j.ijporl.2016.04.024

26. Murhekar M. Epidemiology of diphtheria in India, 1996–2016: implications for prevention and control. Am J Trop Med Hyg. 2017;97:313–8. PubMed https://doi.org/10.4269/ajtmh.17-0047

27. Lodeiro-Colatosti A, Reischl U, Holzmann T, Hernández-Pereira CE, Rísquez A, Paniz-Mondolfi AE. Diphtheria outbreak in Amerindian communities, Wonken, Venezuela, 2016–2017. Emerg Infect Dis. 2018;24:1340–4. PubMed https://doi.org/10.3201/eid2407.171712

28. Sein C, Tiwari T, Macneil A, Wannemuehler K, Soulaphy C, Souliphone P, et al. Diphtheria outbreak in Lao People's Democratic Republic, 2012–2013. Vaccine. 2016;34:4321–6. PubMed https://doi.org/10.1016/j.vaccine.2016.06.074

29. Sadoh AE, Oladokun RE. Re-emergence of diphtheria and pertussis: implications for Nigeria. Vaccine. 2012;30:7221–8. PubMed https://doi.org/10.1016/j.vaccine.2012.10.014

30. Lumio J, Suomalainen P, Olander RM, Saxén H, Salo E. Fatal case of diphtheria in an unvaccinated infant in Finland. Pediatr Infect Dis J. 2003;22:844–6. PubMed https://doi.org/10.1097/01.inf.0000083906.24285.23

31. Nandi R, De M, Browning S, Purkayastha P, Bhattacharjee AK. Diphtheria: the patch remains. J Laryngol Otol. 2003;117:807–10. PubMed https://doi.org/10.1258/002221503770716250

32. Krumina A, Logina I, Donaghy M, Rozentale B, Kravale I, Griskevica A, et al. Diphtheria with polyneuropathy in a closed community despite receiving recent booster vaccination. J Neurol Neurosurg Psychiatry. 2005;76:1555–7. PubMed https://doi.org/10.1136/jnnp.2004.056523

33. Prasad KC, Kaniyur V, Shenoy S, Prasad SC. Upper respiratory tract and cutaneous diphtheria. Indian J Otolaryngol Head Neck Surg. 2005;57:250–2. PubMed

34. Sharma NC, Banavaliker JN, Ranjan R, Kumar R. Bacteriological and epidemiological characteristics of diphtheria cases in and around Delhi: a retrospective study. Indian J Med Res. 2007;126:545–52. PubMed

35. Communicable disease and health protection quarterly review: July to September 2008. J Public Health (Oxf). 2008;30:510–1. PubMed https://doi.org/10.1093/pubmed/fdn090

36. Bitragunta S, Murhekar MV, Hutin YJ, Penumur PP, Gupte MD. Persistence of diphtheria, Hyderabad, India, 2003–2006. Emerg Infect Dis. 2008;14:1144–6. PubMed https://doi.org/10.3201/eid1407.071167

37. Fajolu IB, Egre-Okwaji MT. Sporadic emergence of probable cases of diphtheria. Nigerian Journal of Paediatrics. 2009;36:87–90.

38. Fredlund H, Norén T, Lepp T, Morfeldt E, Henriques Normark B. A case of diphtheria in Sweden, October 2011. Euro Surveill. 2011;16:20038. PubMed

39. Mattos-Guaraldi AL, Damasco PV, Gomes DL, Melendez MG, Santos LS, Marinelli RS, et al. Concurrent diphtheria and infectious mononucleosis: difficulties for management, investigation and control of diphtheria in developing countries. J Med Microbiol. 2011;60:1685–8. PubMed https://doi.org/10.1099/jmm.0.027870-0

40. Rousseau C, Belchior E, Broche B, Badell E, Guiso N, Laharie I, et al. Diphtheria in the south of France, March 2011. Euro Surveill. 2011;16:pii:19867. PubMed

41. Lindhusen-Lindhé E, Dotevall L, Berglund M. Imported larynegeal and cutaneous diphtheria in tourists returning from western Africa to Sweden. Euro Surveill. 2012;17:pii:21089.

42. Paul NI, Ugwu RO. Diphtheria in a 13 year old adolescent girl: management challenges. Niger J Paediatr. 2014;41:247–50. https://doi.org/10.4314/njp.v41i3.19

43. Garib Z, Danovaro-Holliday MC, Tavarez Y, Leal I, Pedreira C. Diphtheria in the Dominican Republic: reduction of cases following a large outbreak. Rev Panam Salud Publica. 2015;38:292–9. PubMed

44. Sane J, Sorvari T, Widerström M, Kauma H, Kaukoniemi U, Tarkka E, et al. Respiratory diphtheria in an asylum seeker from Afghanistan arriving to Finland via Sweden, December 2015. Euro Surveill. 2016;21:doi: 10.2807/1560-7917.ES.2016.21.2.30105. PubMed https://doi.org/10.2807/1560-7917.ES.2016.21.2.30105

45. Bhagat S, Grover SS, Gupta N, Roy RD, Khare S. Persistence of *Corynebacterium diphtheriae* in Delhi and National Capital Region (NCR). Indian J Med Res. 2015;142:459–61. PubMed https://doi.org/10.4103/0971-5916.169212

46. Basak M, Chaudhuri SB, Ishore K, Bhattacherjee S, Das DK. Pattern and trend of morbidity in the infectious disease ward of North Bengal Medical College and Hospital. J Clin Diagn Res. 2015;9:LC01–04. PubMed https://doi.org/10.7860/JCDR/2015/15202.6741

47. Dandinarasaiah M, Vikram BK, Krishnamurthy N, Chetan AC, Jain A. Diphtheria re-emergence: problems faced by developing countries. Indian J Otolaryngol Head Neck Surg. 2013;65:314–8. PubMed https://doi.org/10.1007/s12070-012-0518-5

48. Pan American Health Organization. Diphtheria outbreak in Paraguay. PAHO Immunization Newsletter. 2002;24:6 [cited 2019 Jul 26]. https://www.paho.org/english/ad/fch/im/sne2403.pdf?ua=1

49. Pan American Health Organization. Diphtheria outbreak in Paraguay: an update. PAHO Immunization Newsletter. 2002;24:7 [cited 2019 Jul 26]. https://www.paho.org/english/ad/fch/im/sne2405.pdf?ua=1

50. Centers for Disease Control and Prevention. Fatal respiratory diphtheria in a U.S. traveler to Haiti—Pennsylvania, 2003. MMWR Morb Mortal Wkly Rep. 2004;52:1285–6. PubMed

51. Death in a child infected with toxigenic *Corynebacterium diphtheriae* in London. London: Public Health England; 2008. No. 19 [cited 2019 Jul 16]. http://www.wales.nhs.uk/sitesplus/888/news/9765

52. Pan American Health Organization. Diphtheria outbreak in Haiti, 2009. PAHO Immunization Newsletter. 2009;31:1 [cited 2019 Jul 27]. http://www1.paho.org/hq/dmdocuments/2010/Sne3106.pdf

53. NNDSS Annual Report Writing Group. Australia's notifiable disease status, 2011: annual report of the National Notifiable Diseases Surveillance System. Commun Dis Intell Q Rep. 2013;37:E313–93. PubMed

54. European Centre for Disease Prevention and Control. A case of diphtheria in Spain: rapid risk assessment. Stockholm: The Centre; 2015 [cited 2019 Jul 16] https://ecdc.europa.eu/en/publications-data/rapid-risk-assessment-case-diphtheria-spain-15-june-2015

55. A fatal case of diphtheria in Belgium. In: Control ECfDPa, editor. Rapid Risk Assessment. Stockholm: ECDC; 2016. p. 1–10 [cited 2019 Jul 16]. https://ecdc.europa.eu/en/publications-data/rapid-risk-assessment-fatal-case-diphtheria-belgium-30-march-2016

56. Pan American Health Organization/World Health Organization. Annual report 2016. Washington (DC): The Organizations; 2017 [cited 2019 Jul 16]. https://www.paho.org/hq/dmdocuments/2016/2016-phe-epi-alerts-updates.pdf

57. Pan American Health Organization/World Health Organization. Epidemiological update: diphtheria. Washington (DC): The Organizations; 2017 [cited 2019 Jul 16]. https://www.paho.org/hq/dmdocuments/2017/2017-dec-15-phe-epi-update-diphtheria.pdf

58. World Health Organization. Diphtheria outbreak, Cox's Bazar, Bangladesh. Geneva: The Organization; 2018 [cited 2019 Jul 16]. https://www.who.int/csr/don/13-december-2017-diphtheria-bangladesh/en/

59. Pan American Health Organization. Diphtheria Outbreak in the Dominican Republic. Washington (DC): The Organization. The PAHO Immunization Newsletter. 2004;26:1 [cited 2019 Jul 26]. https://www.paho.org/english/ad/fch/im/sne2603.pdf?ua=1

60. European Centre for Disease Prevention and Control. The European Surveillance System (TESSy). Stockholm: The Centre; 2006–2017 [cited 2019 Jul 16]. https://ecdc.europa.eu/en/publications-data/european-surveillance-system-tessy
